# A Fast Approach to Removing Muscle Artifacts for EEG with Signal Serialization Based Ensemble Empirical Mode Decomposition

**DOI:** 10.3390/e23091170

**Published:** 2021-09-06

**Authors:** Yangyang Dai, Feng Duan, Fan Feng, Zhe Sun, Yu Zhang, Cesar F. Caiafa, Pere Marti-Puig, Jordi Solé-Casals

**Affiliations:** 1 College of Artificial Intelligence, Nankai University, Tianjin 300350, China; daiyangyang@mail.nankai.edu.cn (Y.D.); nkfengfan@mail.nankai.edu.cn (F.F.); ccaiafa@fi.uba.ar (C.F.C.); 2 Computational Engineering Applications Unit, Head Office for Information Systems and Cybersecurity, RIKEN, Wako 351-0198, Japan; zhe.sun.vk@riken.jp; 3 Department of Bioengineering, Lehigh University Bethlehem, Bethlehem, PA 18015, USA; yuzhang@lehigh.edu; 4 Instituto Argentino de Radioastronomía—CCT La Plata, CONICET/CIC-PBA/UNLP, Villa Elisa 1894, Argentina; 5 Data and Signal Processing Research Group, University of Vic—Central University of Catalonia, 08500 Vic, Catalonia, Spain; pere.marti@uvic.cat

**Keywords:** EEG, EMG artifact rejection, signal serialization, EEMD, CCA

## Abstract

An electroencephalogram (EEG) is an electrophysiological signal reflecting the functional state of the brain. As the control signal of the brain–computer interface (BCI), EEG may build a bridge between humans and computers to improve the life quality for patients with movement disorders. The collected EEG signals are extremely susceptible to the contamination of electromyography (EMG) artifacts, affecting their original characteristics. Therefore, EEG denoising is an essential preprocessing step in any BCI system. Previous studies have confirmed that the combination of ensemble empirical mode decomposition (EEMD) and canonical correlation analysis (CCA) can effectively suppress EMG artifacts. However, the time-consuming iterative process of EEMD may limit the application of the EEMD-CCA method in real-time monitoring of BCI. Compared with the existing EEMD, the recently proposed signal serialization based EEMD (sEEMD) is a good choice to provide effective signal analysis and fast mode decomposition. In this study, an EMG denoising method based on sEEMD and CCA is discussed. All of the analyses are carried out on semi-simulated data. The results show that, in terms of frequency and amplitude, the intrinsic mode functions (IMFs) decomposed by sEEMD are consistent with the IMFs obtained by EEMD. There is no significant difference in the ability to separate EMG artifacts from EEG signals between the sEEMD-CCA method and the EEMD-CCA method (*p* > 0.05). Even in the case of heavy contamination (signal-to-noise ratio is less than 2 dB), the relative root mean squared error is about 0.3, and the average correlation coefficient remains above 0.9. The running speed of the sEEMD-CCA method to remove EMG artifacts is significantly improved in comparison with that of EEMD-CCA method (*p* < 0.05). The running time of the sEEMD-CCA method for three lengths of semi-simulated data is shortened by more than 50%. This indicates that sEEMD-CCA is a promising tool for EMG artifact removal in real-time BCI systems.

## 1. Introduction

Brain–computer interface (BCI) is a type of human–computer interaction, which can provide a possible way to improve the quality of life for the disabled [1,2]. Through the non-muscle information channel, BCI converts the electrophysiological signals collected in the brain into the control commands of external devices to achieve communication between the brain and the external environment [3]. In recent years, as the core of human–machine hybrid intelligence, the research progress of BCI has attracted great attention from academia and industry. The signal acquisition method of non-invasive BCI is safe and simple with the advantage of avoiding surgery. With its highly accurate time resolution and excellent clinical environment applicability, the electroencephalogram (EEG) has become the main non-invasive neurophysiological recording technology used by BCI control systems to monitor brain consciousness activities [4].The EEG signal has low amplitude and high time-varying characteristics. During the acquisition process, EEG is often mixed with various artifacts generated by non-cerebral nerve tissues, such as electrooculograms, electromyograms (EMGs), electrocardiograms, and power frequency interference [5]. These interference signals and EEG signals are overlapped with each other, submerging the original waveform characteristics of EEG signals. Therefore, EEG denoising is indispensable [6,7,8]. The effect of noise rejection directly affects the performance of the BCI system. Among the common artifacts, EMG is usually the most difficult to eliminate due to its high amplitude, wide frequency domain, and variable spatial distribution [9]. In consideration of the complex physiological process and the insufficient prior knowledge for EMG, blind source separation (BSS) technology is often recommended to separate the EMG noise from EEG signals [10].

Independent component analysis (ICA) is a BSS algorithm widely used in EEG signal denoising [11]. ICA separates statistically independent signals from multi-channel data with unknown sources exploiting high-order statistics. Then, the components identified as artifacts are removed. The clean EEG data are reconstructed from the retained components. Generally, ICA can eliminate the artifacts with fixed spatial distribution. The amplitude and shape of EMG artifacts depend on the contraction degree, the type, and the quantity of muscle. More importantly, the spatial distribution of EMG artifacts is variable. Related studies indicate that the EMG artifacts in EEG signals are not effectively identified by means of ICA [12,13]. Subsequently, canonical component analysis (CCA) is proposed as an alternative method [14]. The original EEG data and their temporally delayed version are designated as the first and second datasets, respectively. Using second-order statistics, CCA extracts the sources from the signals. These sources have the largest autocorrelation coefficients and are not correlated with each other. Compared with EEG, the temporal characteristic of EMG is more similar to that of white noise. In other words, EMG has a relatively low autocorrelation coefficient. Owing to this unique feature, the sources with an autocorrelation coefficient lower than a reasonable threshold are considered as EMG artifacts and successfully isolated from EEG. Nevertheless, BSS algorithms such as ICA or CCA may still be unable to completely distinguish non-brain sources from brain sources. For example, a low signal-to-noise ratio (SNR), complex contamination, and the number of available channels (less than the number of sources) will increase the processing difficulty of traditional BSS technology.

The combination of single-channel decomposition and BSS technology is confirmed to have a significant effect on the suppression of artifacts. Zeng et al. [15] explored the ability of ensemble empirical mode decomposition (EEMD) and ICA (EEMD-ICA) to recover noisy multi-dimensional EEG data. In their work, the EEG signal of each channel is decomposed into a finite number of intrinsic mode functions (IMFs) employing EEMD. Then, the artifact-like IMFs of all channels are screened out. The artifacts scattered on these IMFs are concentrated on a few components adopting ICA. Finally, these artifacts are removed, and the clean multidimensional EEG data are reconstructed. The results show that in terms of the normalized mean square error and the structural similarity, EEMD-ICA is superior to the two main noise rejection methods (ICA and wavelet ICA [16]), especially in the case of low SNR. Chen et al. [17] adequately considered the temporal structural characteristics of EMG. Based on the work of Zeng et al., they proposed a new method that combines EEMD with CCA (EEMD-CCA) to remove EMG artifacts in EEG data. The test results on simulated, semi-simulated, and real data showed that, compared with the current state-of-the-art technology (ICA, CCA and EEMD-ICA), the EEMD-CCA method has more outstanding reliability. Even if the SNR is less than 2 dB, the EEMD-CCA method can also maintain good performance. With the popularization of home health care monitoring, EEG equipment has shifted to installing a small number of electrodes for ease of use [18]. Through setting the different number of channels, the effectiveness of EEMD-CCA under few-channel settings was also verified. In addition, Mucarquer et al. [19] proposed an extended EEMD-CCA method to help eliminate EMG artifacts by using an EMG array as information.

In practical applications, the final IMFs obtained by EEMD need a large number of iterations. In each iteration, the upper and lower envelopes are found by searching for the extreme points and the coefficients in each spline curve equation. This process is time-consuming, which makes it difficult for the EEMD-CCA method to meet the monitoring requirements of BCI in real time. On the premise of maintaining the existing algorithm, Zhang et al. [20] proposed a feasible optimization scheme by changing the structure of the input signal. With a simple signal serialization, multi-channel signals are concatenated in series into a single one-dimensional signal. Under this cascade mode, EEMD realizes synchronous decomposition of multi-channel signals. Furthermore, the signal serialization based EEMD (sEEMD) can improve the speed of signal decomposition.

In this paper, a method combining sEEMD and CCA is proposed to remove EMG artifacts from EEG signals, namely sEEMD-CCA. The rest of this article is arranged as follows. In the Section 2, the principles of sEEMD-CCA and the two state-of-the-art technologies used to remove EMG artifacts are introduced. The Section 3 describes the datasets used and the corresponding evaluation measures for denoising performance in detail. In the Section 4, the denoising performance and running time of the sEEMD-CCA method are mainly discussed. Section 5 is an in-depth summary of this research.

## 2. Materials and Methods

### 2.1. EEMD

Empirical mode decomposition (EMD) is an adaptive data processing or mining method [21]. The algorithm can decompose the complex signal into a series of IMFs containing local feature information at different time scales to represent the original signal. The IMFs have the following two characteristics: (1) in the entire data range, the number of extreme values and the number of zero crossings differ by one at maximum, and (2) the mean value of envelopes defined by local maxima and local minima is zero at any time. Due to the intermittent phenomenon, the IMFs obtained based on EMD have the problem of modal aliasing. EEMD [22] is an improved method of EMD for modal aliasing. The basic idea is as follows.

The white noise $n_{i}\left(t\right)$ with a mean value of 0 and a constant variance is added to the original signal x(t) to obtain *m* new time series,

(1) $$x_{i}\left(t\right)=x\left(t\right)+n_{i}\left(t\right),i=1,2,\cdots ,m.$$

The *m* signals are decomposed into IMFs utilizing EMD,

(2) $$x_{i}\left(t\right)=\sum_{j=1}^{k}imf_{ij}\left(t\right)+r_{i}\left(t\right),i=1,2,\cdots ,m,j=1,2,\cdots ,k,$$

where $imf_{ij}\left(t\right)$ represents the *j*-th IMF of the *i*-th signal, and $r_{i}\left(t\right)$ is the residual amount of the *i*-th signal.

According to the zero-mean characteristic of white noise, the mean values of the IMFs and the residual amount obtained by m-time decomposition are calculated to eliminate the influence of adding white noise to the true IMFs. The final result of EEMD is as follows,

(3) $$imf_{j}=\frac{1}{m}\sum_{i=1}^{m}imf_{ij}\left(t\right),r\left(t\right)=\frac{1}{m}\sum_{i=1}^{m}r_{i}\left(t\right),i=1,2,\cdots ,m,j=1,2,\cdots ,k.$$

Prior to executing the EEMD algorithm, it is necessary to preset the ensemble number and the standard deviation of the added white noise amplitude. The ensemble number is the number of times that white noise is added. A big ensemble number seriously increases the computational cost of EEMD, while a small ensemble number will make the decomposition effect of EEMD worse. Previous studies have pointed out that if the ensemble number exceeds 10, the decomposition effect has little difference from the increase of the ensemble number [17,23]. Therefore, in consideration of the decomposition effect and computational cost, the ensemble number was set to 25 in this study. The amplitude of white noise also closely affects the decomposition effect of the EEMD algorithm. Based on experience, the recommended standard deviation of the white noise amplitude was set to 0.2 times the signal standard deviation [22]. For different application scenarios, please refer to the relevant references for more details on the parameter selection of the ensemble number and the amplitude of white noise.

### 2.2. Serial EEMD

#### 2.2.1. The Serialization of Two-Channel Signals

**Figure 1:**
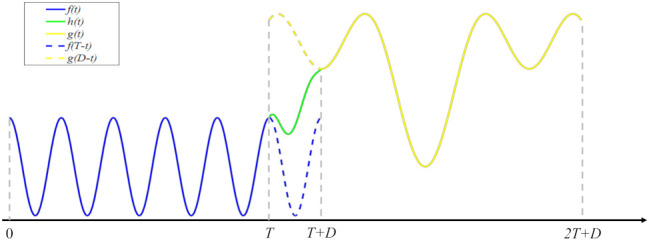
Schematic diagram of two-channel signal serialization.

Here, we first introduce two-channel signal serialization, based on the procedure described in [20]. The schematic diagram is shown in Figure 1. As can be seen, a transition signal is embedded between the two signals in order to avoid discontinuity at the joint. In detail, at the tail of one signal and the head of the other signal, a small segment of the signal with equal length is respectively intercepted and flipped upside down. Then, a transition signal is constructed based on the two flipped signals to concatenate the two original signals. The specific mathematical expression is as follows.

f(t), g(t) represent two signals of length *T*. Generally, f(T)≠g(0). h(t) represents a transition signal of length *D* (*D* < *T*). h(t) is simply defined as,

(4) $$h\left(t\right)=\left(1-\frac{t}{D}\right)f\left(T-t\right)+\frac{t}{D}g\left(D-t\right),t\in \left[0,D\right],$$

where f(T−t) and g(D−t) are two flipped signals of length *D*. Particularly, h(0)=f(T), h(D)=g(0).

Thus, the serialized signal is,

(5) $$s\left(t\right)=\left\{\begin{array}{c} \begin{array}{cc} f(t) & ,t\in [0,T] \end{array}\\ \begin{array}{cc} h(t-T) & ,t\in [T,T+D] \end{array}\\ \begin{array}{cc} g(t-T-D) & ,t\in [T+D,2T+D] \end{array} \end{array}\right..$$

It is easy to verify that s(t) is continuous and relatively smooth at the joint.

#### 2.2.2. The Serialization of Multi-Channel Signals

**Figure 2:**
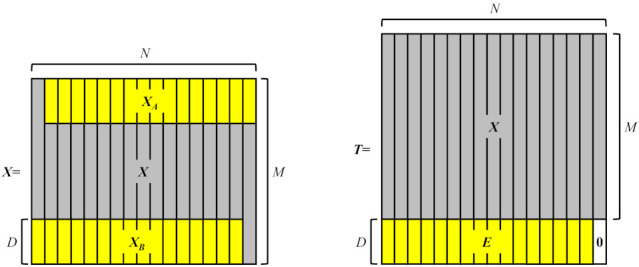
Descriptions of the serialization algorithm applied to multi-channel signals.

The serialization process of multi-channel signals is adequately explained in Figure 2. First, it is necessary to define the transition signals of length *D* embedded between multi-channel signals. For convenience, the mathematical descriptions of the serialization process are expressed in the form of the matrices or vectors. *N* signals of length *M* are denoted as $X_{M\times N}=\left(x_{1},x_{2},\cdots ,x_{N}\right)$, where $x_{i}$ (i=1,2,⋯,N) is an *M*-dimensional vector. The submatrix $X_{A}=X_{1:D,2:N}$ is the head of some signals, while the submatrix $X_{B}=X_{M-D+1:M,1:N-1}$ corresponds to the tail of some signals. The transition signal can be expressed as follows,

(6) $$E_{D\times (N-1)}={X_{A}}^{f}⊙\left(au^{T}\right)+{X_{B}}^{f}⊙\left(a^{f}u^{T}\right),$$

where *E* is the matrix manifestation of the transition signals, *a* is a *D*-dimensional vector, ai=ii(D+1)(D+1), i=1,2,⋯,D, *u* is an *N* − 1 dimensional vector of all ones, the symbol ⊙ is the Hadamard product operator, the superscript *T* indicates transposition, and the superscript *f* represents flipping the vector upside down.

By filling a *D*-dimensional all zero vector *z*, a matrix containing the original signals and the transition signals can be generated,

(7) $$T_{\left(M+D\right)\times N}=\left[\begin{array}{c} X_{M\times N}\\ \begin{array}{cc} E_{D\times \left(N-1\right)} & z_{D\times 1} \end{array} \end{array}\right].$$

Subsequently, the matrix *T* is vectorized,

(8) $$t_{\left(MN+DN\right)\times 1}=vec\left(T\right).$$

Finally, the *D* zeros in the vector *t* are eliminated. The serialized signal obtained is,

(9) $$x_{\left(MD+DN-D\right)\times 1}=t_{1:\left(MN+DN-D\right)}.$$

#### 2.2.3. The IMFs Reconstruction for the Original Signals

The mathematical model of signal decomposition is,

(10) $$R_{(MN+DN-D)\times K}=F\left(x_{(MN+DN-D)\times 1}\right),$$

where F(·) represents the operator that performs EEMD. *K* represents the number of IMFs. In this process, the transition signals embedded in the original signals are also decomposed. The corresponding decomposition results are stored.

In order to obtain the IMFs of each channel, the matrix *R* is expanded using an all-zero matrix $Z_{D\times K}$,

(11) $$S_{(MN+DN-D)\times K}=\left[\begin{array}{c} R_{\left(MN+DN-D\right)\times K}\\ Z_{D\times K} \end{array}\right].$$

**Figure 3:**
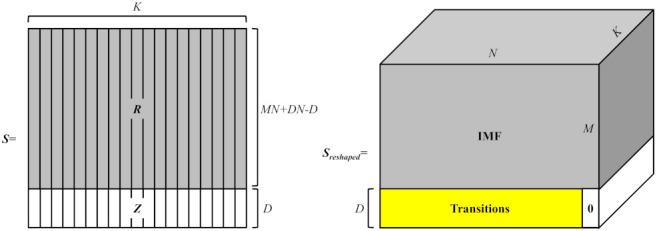
IMFs reconstruction algorithm for multi-channel signals.

Then, the matrix *S* is reshaped into $S_{reshaped}$ with the size of (M+D)×N×K, as shown in Figure 3. By removing the decomposition results of the transition parts, the final IMFs can be obtained,

(12) $$IMF_{M\times N\times K}={S_{reshaped}}_{{}_{1:M,1::N,1:K}}.$$

### 2.3. CCA

$X=\left(x_{1},x_{2},\cdots ,x_{N}\right)\in R^{P\times N}$ and $Y=\left(y_{1},y_{2},\cdots ,y_{N}\right)\in R^{Q\times N}$ are two zero-centered datasets. The aim of CCA is to seek the vectors $w_{x}\in R^{P}$ and $w_{y}\in R^{Q}$ employing the optimal correlation criterion so that the correlation coefficient between the synthetic variables $u=w_{x}^{T}X$ and $v=w_{y}^{T}Y$ is maximal [14]. The concrete optimization problem can be expressed as follows,

(13) $$\max_{w_{x},w_{y}}\rho =\max_{w_{x},w_{y}}\frac{w_{x}^{T}c_{xy}w_{y}}{\sqrt{w_{x}^{T}c_{xx}w_{x}w_{x}^{T}c_{yy}w_{y}}},$$

where $C_{xx}=XX^{T}$ and $C_{yy}=YY^{T}$ are the autocovariance matrices of *X* and *Y*, respectively. $C_{xy}=XY^{T}$ is the covariance matrix of *X* and *Y*.

In the actual solution, the correlation coefficient ρ depends on the direction of $w_{x}$ and $w_{y}$, independent of their length. Let $w_{x}^{T}C_{xx}w_{x}=1$, $w_{x}^{T}C_{yy}w_{y}=1$. Then, the objective function is equivalently represented as,

(14) $$\left\{\begin{array}{c} \max_{w_{x},w_{y}}\rho =\max_{w_{x},w_{y}}w_{x}^{T}c_{xy}w_{y}\\ s.t.\left\{\begin{array}{c} w_{x}^{T}C_{xx}w_{x}=1\\ w_{x}^{T}C_{yy}w_{y}=1 \end{array}\right. \end{array}\right..$$

To solve this problem, a function is defined by the Lagrange multiplier method,

(15) $$L\left(\lambda_{1},\lambda_{2},w_{x},w_{y}\right)=w_{x}^{T}C_{xy}w_{y}-\frac{\lambda_{1}}{2}\left(w_{x}^{T}C_{xx}w_{x}-1\right)-\frac{\lambda_{2}}{2}\left(w_{y}^{T}C_{yy}w_{y}-1\right).$$

The derivatives of function $L\left(\lambda_{1},\lambda_{2},w_{x},w_{y}\right)$ with respect to $w_{x}$ and $w_{y}$ are calculated and set to 0, separately,

(16) $$\left\{\begin{array}{c} \frac{\partial L}{\partial w_{x}}=C_{xy}w_{y}-\lambda_{1}C_{xx}w_{x}=0\\ \frac{\partial L}{\partial w_{y}}=C_{yx}w_{x}-\lambda_{2}C_{yy}w_{y}=0 \end{array}\right..$$

From Formula (16), it is easy to know that $\lambda_{1}=\lambda_{2}=\lambda$. Based on the assumption that λ≠0, $C_{xx}$ and $C_{yy}$ are non-singular matrices, Formula (17) can also be obtained,

(17) $$\left\{\begin{array}{c} C_{xy}C_{yy}^{-1}C_{yx}w_{x}=\lambda^{2}C_{xx}w_{x}\\ C_{yx}C_{xx}^{-1}C_{xy}w_{y}=\lambda^{2}C_{yy}w_{y} \end{array}\right..$$

Formula (17) transforms the problem of seeking the maximum value into a problem of solving the generalized eigenvalues and eigenvectors. In general, the eigenvectors corresponding to the largest $r=\min\left(P,Q\right)$ eigenvalues are taken as $w_{x_{i}}$ and $w_{y_{i}}$ (i=1,2,⋯,r) in turn. Consequently, $u_{i}=w_{x_{i}}^{T}X$, $v_{i}=w_{y_{i}}^{T}Y$ (i=1,2,⋯,r). $u_{i}$ and $v_{i}$ are called canonical variates of *X* and *Y*. *U* and *V* include all of the canonical variates of each dataset. The relationship between the original datasets and the canonical variates can be indicated as follows, $U=W_{x}^{T}X$, $V=W_{y}^{T}Y$, with $W_{x}=\left(w_{x_{1}},w_{x_{2}},\cdots ,w_{x_{r}}\right)$ and $W_{y}=\left(w_{y_{1}},w_{y_{2}},\cdots ,w_{y_{r}}\right)$. If $C_{xx}$ and $C_{yy}$ are singular matrices, first regularize $C_{xx}$ and $C_{yy}$, and then invert them. That is, $C_{xx}+\alpha I$ and $C_{yy}+\beta I$ are used to replace $C_{xx}$ and $C_{yy}$. α and β are regularization parameters.

### 2.4. ICA

ICA is a data-driven method based on high-order statistical characteristics of signals, which separate independent sources from the mixed observation signals without any prior knowledge [24]. The classic description is as follows, X(t)=AS(t), where $X\left(t\right)={\left[x_{1}\left(t\right),x_{2}\left(t\right),\cdots ,x_{m}\left(t\right)\right]}^{T}$ is *m* random observation signals. $S\left(t\right)={\left[s_{1}\left(t\right),s_{2}\left(t\right),\cdots ,s_{n}\left(t\right)\right]}^{T}$ represents *n* independent sources. *A* is a mixed matrix with the size of *m* × *n*. Both the mixed matrix and the sources are unknown. The goal of ICA is to find an unmixed matrix *W* such that the following relationship holds, Y=WX(t)=WAS(t). The components of *Y* are independent of each other and are as close as possible to the sources.

### 2.5. sEEMD-CCA Method for EMG Artefact Rejection

**Figure 4:**
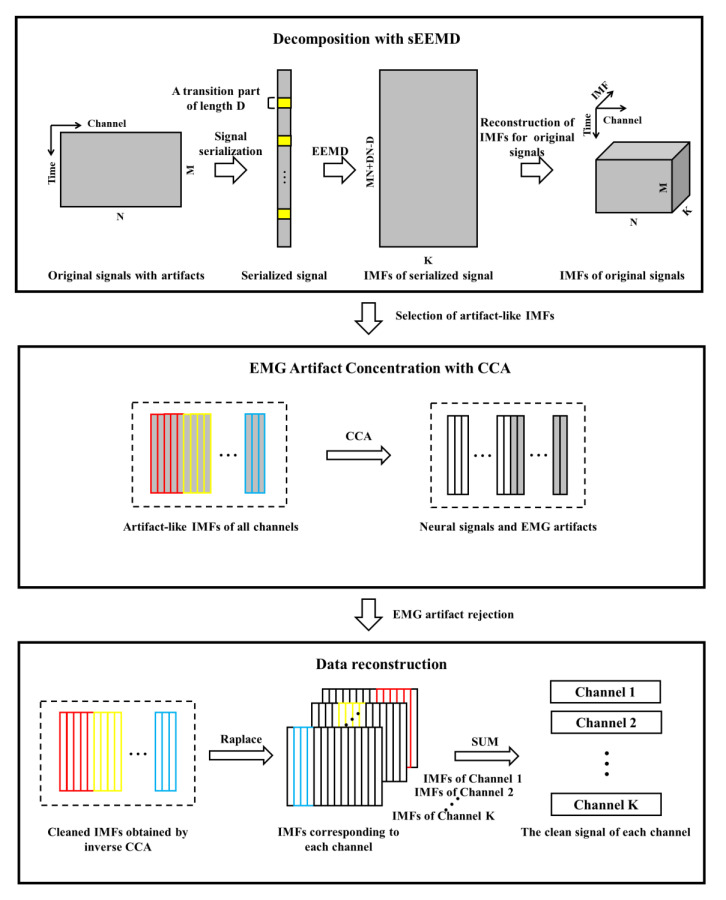
Technological process of the sEEMD-CCA method to remove EMG artifacts.

The process of the sEEMD-CCA method to remove EMG artifacts is described in detail in Figure 4. This method is mainly divided into three steps.

In the first step, EEG signals $X_{M\times N}=\left(x_{1},x_{2},\cdots ,x_{N}\right)$ contaminated by EMG artifacts are decomposed into a series of IMFs employing sEEMD, where *M* is the length of a signal. *N* is the number of channels. At this stage, *N*-channel signals are concatenated into a one-dimensional signal using signal serialization. Then, EEMD decomposes the serialized signal into several IMFs. After reshaping signal, the IMFs of all channels represented by $IMF_{original}$ can be separated. Part of these extracted IMFs are a mixture of EEG activity and EMG artifacts. Therefore, before the next step, it is important to determine the appropriate predefined purification rule. EMG has a relatively lower autocorrelation coefficient, behaving more like the temporal structure characteristic of white noise than EEG. We calculate the autocorrelation coefficients of all IMFs. According to the unique property of EMG, an IMF with an autocorrelation coefficient lower than the reasonable threshold is identified as an artifact component. In this study, the threshold was empirically set to 0.9. More information about threshold selection can be found in the work [17]. The selected IMFs are reorganized together, denoted as $IMF_{artifact}$.

In the second step, the EMG artifacts scattered on IMFs are concentrated on a few components based on CCA. $IMF_{artifact}$ and its version with a time delay of 1 unit are respectively designated as the first and second datasets. CCA solves the sources of the mixed signal by maximizing the correlation coefficient between the linear combinations of two datasets. *U* and *V* are the sources of $IMF_{artifact}$ and its delayed versions. They respectively contain all linear combinations of each dataset, and their own components are mutually uncorrelated. Similarly, with the knowledge of the low autocorrelation coefficient, the EMG artifact component in *U* is set to zero.

In the third step, clean EEG data are reconstructed by summing up all of the artifact-free IMFs of each channel. Inverse CCA is applied to *U* to obtain cleaned IMFs, which are stored in the matrix $IMF_{clean}$. Subsequently, the artifact component in $IMF_{original}$ is replaced by the cleaned IMF at the corresponding position in $IMF_{clean}$. The clean EEG data of each channel are recovered from the IMFs of $IMF_{original}$.

### 2.6. EEMD-BSS Methods for EMG Artifact Rejection

EEMD is an adaptive data-driven method to effectively explore the structure of neural data. EEMD decomposes complex data into a series of IMFs that accurately reflect the characteristics of the original data. It has been proven that the combination of EEMD and BSS has better denoising performance in comparison to the traditional BSS technology. EEMD-ICA and EEMD-CCA are two popular EEMD-BSS methods. In the EEMD-ICA method, the multi-channel noisy EEG data are first decomposed into several IMFs by EEMD. The EMG artifact-like components are screened out from the IMFs. Then, ICA is performed on these components, and the EMG artifacts hidden in the selected IMFs are concentrated on a small part of the components. Finally, these artifacts are eliminated to reconstruct the artifact-free IMFs. Clean EEG data are obtained by summing up all of the clean IMFs of each channel. The specific details can be found in the work [15]. The EEMD-CCA method to remove EMG artifacts is basically consistent with the EEMD-ICA method. More details are explained in the work [17].

## 3. Data Description and Evaluation Measures

### 3.1. Clean EEG

In order to further verify the effectiveness of the sEEMD-CCA method, semi-simulated data were artificially generated; they are a superimposition of clean EEG and EMG signals. In this study, the Dataset 2a of BCI Competition IV was employed [25]. Twenty-two Ag/AgCl electrodes (Fz, FC3, FC1, FCz, FC2, FC4, C5, C3, C1, Cz, C2, C4, C6, CP3, CP1, CPz, CP2, CP4, P1, Pz, P2, and POz) and three EOGs were used to record the EEG data of four different motor imagery tasks (left hand, right hand, both feet, and tongue) from nine subjects. All signals were collected monopolarly. The left mastoid and the right mastoid served as reference and ground, respectively. The sensitivity of the amplifier was 100 µV. The sampling frequency was 250 Hz. All signals were band-pass filtered between 0.5 and 30 Hz.

The EEG data of each subject contain a total of 288 trials, 6 s for each trial. A visual inspection for all trials was carried out by an experienced neurophysiologist. The artifact label information for each trial can be acquired from h.ArtifactSelection. h.ArtifactSelection is provided by the BCI Competition, which contains a list of zeros and ones. Zero corresponds to a clean trial, whereas one corresponds to a trial with artifacts. We eliminated trials containing artifacts and three EOG electrodes. Two trials of each motor imagery task from each subject were randomly selected to form 12 s EEG data.

### 3.2. Clean EMG

Nine healthy subjects (seven males and two females, aged 22 to 25 years old) participated in the EMG acquisition experiment. Each of them was not familiar with the specific content of the whole process and was required to complete five movements (namely hand closing, hand opening, wrist extension, wrist flexion, and forefinger pointing) successively. Each movement lasted for 6 s, followed by a 4 s rest. The clean EMG signals were gathered from both sides of the right forearm with the surface EMG system Telemyo DTS (Noraxon, USA). To match with the EEG data, the sampling frequency of the EMG recordings was set to 250 Hz. Then, the next EMG collection started after one minute of rest. All operations were repeated until 10 sets of data were obtained for each subject.

For the same movement of each subject, 10 signals collected by the same electrode were stored together. Therefore, there were a total of 45 such pieces of data for each acquisition position. We randomly selected 22 pieces of data from a channel. From each of them, two recordings were randomly screened out to generate 12 s EMG data. Thus, 22-channel EMG signals were obtained. This process was completed 10 times.

### 3.3. The Semi-Simulated Data

The semi-simulated data were constructed in accordance with the following method,

(18) $$X=X_{EEG}+\lambda \cdot X_{EMG},$$

where $X_{EEG}$ and $X_{EMG}$ are 22-channel EEG and 22-channel EMG data, respectively, and λ is the parameter that controls the SNR.

The relationship between λ and SNR is,

(19) $$SNR=\frac{RMS(X_{EEG})}{RMS(\lambda \cdot X_{EMG})},$$

where RMS(·) is the operator used to calculate the root mean squared (RMS). RMS is expressed as

,
(20) $$RMS\left(X\right)=\sqrt{\frac{1}{M\cdot N}\sum_{m=1}^{M}\sum_{n=1}^{N}X^{2}\left(m,n\right)},$$

where *M* represents the number of channels and *N* denotes the number of sampling points.

**Figure 5:**
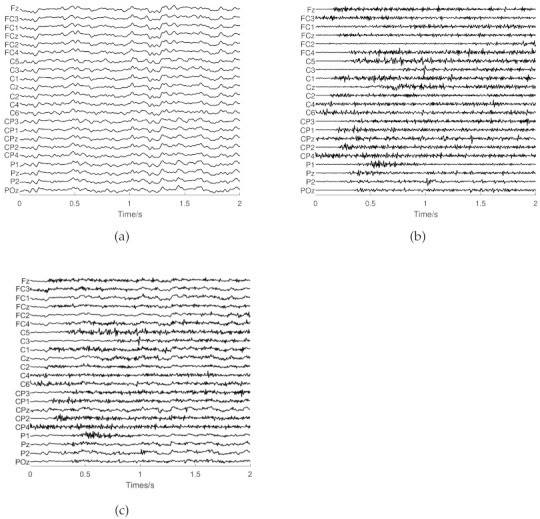
Examples of data with 2 s length. (**a**) The clean EEG. (**b**) The clean EMG. (**c**) The semi-simulated data with an SNR of 0.5 dB.

In this study, the SNR was taken from 0.5 to 5.5 dB at the step of 0.5 dB. Examples of clean EEG, clean EMG, and semi-simulated data are shown in Figure 5.

### 3.4. The Evaluation Measures

We used the relative root mean squared error (RRMSE) and the average correlation coefficient (average CC) to verify the EMG artifact removal effect of each method. RRMSE is an indicator employed to measure the difference between the clean EEG data and the denoised EEG data. The RRMSE is given by,

(21) $$RRMSE=\frac{RMS(X_{EEG}-{\tilde{X}}_{EEG})}{RMS(X_{EEG})},$$

where ${\tilde{X}}_{EEG}$ is the denoised EEG data.

The correlation coefficient reflects the ability of the EEG data with EMG artifacts removed to retain the information of the original EEG data. The correlation coefficients between each channel of the original EEG data and their counterparts were calculated. Average CC is the mean value of these correlation coefficients, regarded as a criterion to judge the similarity between the clean data and the denoised EEG data.

## 4. Results and Discussion

**Figure 6:**
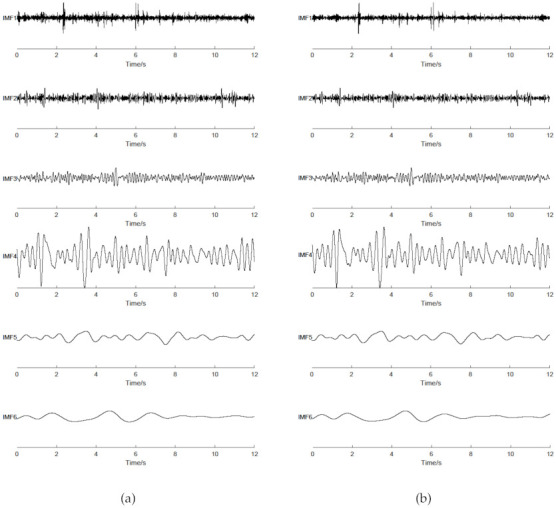
IMFs obtained by EEMD and sEEMD. (**a**) EEMD. (**b**) sEEMD.

EEMD is a classical single-channel decomposition algorithm. In practical applications, the multi-channel EEG signals are used as the control signals of the BCI system. In this case, EEMD decomposes the signal of each channel one by one to realize the decomposition of multi-channel signals. The sEEMD algorithm provides a method for synchronous cascading analysis of multi-channel data, breaking the limitation that a single channel decomposition algorithm can only process a one-dimensional signal at a time. However, whether IMFs decomposed by sEEMD are consistent with those generated by EEMD requires further verification. For this purpose, the first six IMFs of the same channel EEG signal obtained by EEMD and sEEMD are given in Figure 6. Taking frequency and amplitude as the evaluation criteria, the IMFs obtained by the two methods are similar from the high-frequency to the low-frequency ranges. This confirms that sEEMD has an analogous ability to decompose signals to that of EEMD.

Using EEMD and sEEMD to decompose EEG signals, the number of IMFs for the same channel may be different. To be exact, the input signals of EEMD and the input signals of sEEMD are different in the decomposition process of multi-channel signals. This may be the cause for the phenomenon. In each iteration of EMD, the maximum and minimum envelopes of the signal are calculated employing cubic spline interpolation. If a signal generated by subtracting the mean value of the maximum and minimum envelopes from one signal meets the two characteristics of the IMF, it means that this signal is an IMF. The input signal of sEEMD is constructed by embedding transition signals between multiple signals to smoothly concatenate these signals in series. This may have a certain influence on the calculation of the envelopes. Eventually, the number of IMFs obtained by sEEMD may be different from the number of IMFs generated by EEMD when the same signal is decomposed. The concrete mathematical deduction and proof will be discussed in the future. In addition, due to the random nature of EEG signals, there are some differences in the number of the IMFs obtained by EEMD for the different channels. sEEMD decomposes a one-dimensional signal, which is generated by the serialization of multi-channel signals. In the process of IMF reconstruction, the same number of IMFs is allocated to each channel.

**Figure 7:**
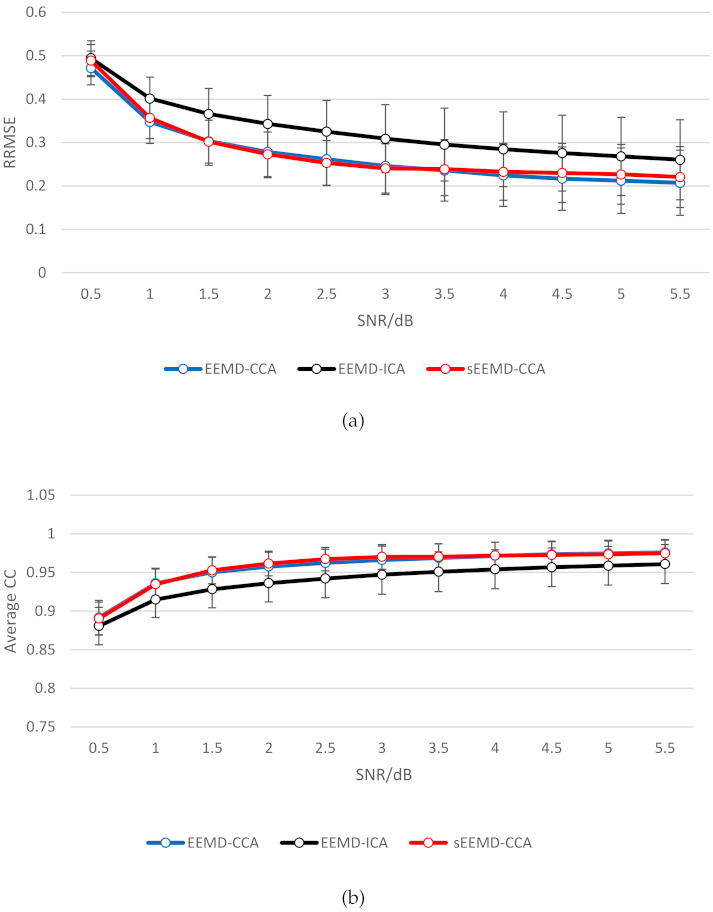
Comparative analysis of denoising performance at an SNR of 0.5 to 5.5 dB. (**a**) RRMSE. (**b**) Average CC.

We conducted a comparative analysis for the denoising performance of the sEEMD-CCA and the two EEMD-BSS methods based on the semi-simulated data. At each SNR value, the 36 clean EEG data from 9 subjects were superimposed on the 10 independent EMG data. Thus, the 360 independent realizations were implemented to evaluate the average performance with the standard deviation of each method. A *t*-test was performed to investigate whether the performance of the methods we compared was statistically significant under various SNR values. The real denoising performance of each method as SNR changes is shown in Figure 7. The specific values of RRMSE and Average CC at each SNR are listed in Table 1 and Table 2. Compared with the EEMD-ICA method, the combination of EEMD and CCA had a better effect in removing EMG artifacts in terms of RRMSE and average CC as evaluation indicators (*p* < 0.05). Even in the case of heavy contamination (SNR < 2 dB), the RRMSE was about 0.3, and the average CC remained above 0.9. These findings are consistent with existing research results [17]. The denoising performance of the sEEMD-CCA method and EEMD-CCA method almost coincided at all SNR values. There was no significant difference in performance between the two methods (*p* > 0.05). This confirms the effectiveness of our proposed method. Furthermore, it should be pointed out that the previous research results showed that the RRMSE between EEG data removing EMG artifacts with EEMD-ICA or EEMD-CCA and the original clean EEG data was almost 0 when the SNR was approximately 4.5 dB. In this study, the minimum RRMSE at an SNR of 5.5 dB was about 0.2. We speculate that this may be related to the EEG or EMG signal used to construct the semi-simulated data.

**Figure 8:**
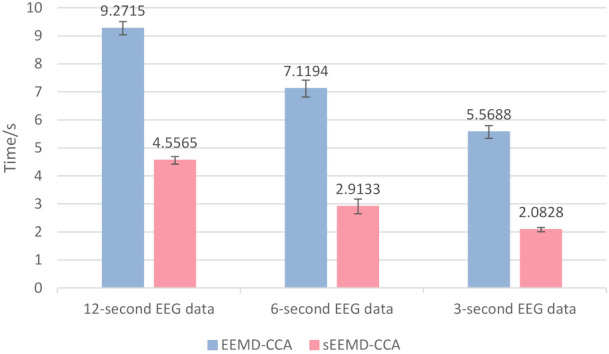
Average decomposition times of sEEMD and EEMD.

In order to confirm that sEEMD-CCA significantly improves the running speed, the computational cost of sEEMD-CCA and EEMD-CCA was analyzed. The test was carried out in MATLAB R2019a (MathWorks Inc., Novi, MI, USA) under Microsoft Windows 10 × 64 OS on a computer with Intel(R) Core (TM) i7-5500U 2.40 GHz CPU and 8.00 GB RAM. There was no parallel computing setting. Prior to this, all analyses were based on 22-channel EEG data with a duration of 12 s, which contain two trials. Here, we provide two additional types of EEG data, with lengths of 6 and 3 s. The former intercepts a complete trial, while the latter only records the mental activities of the subject when he or she performs a motor imagination task. There are 36 segments for each type of EEG data. They are superimposed on 10 EMG data of the same length to construct the semi-simulated data according to an SNR of 0.5 to 5.5 dB. The three types of semi-simulated data were used to examine the dependence of the decomposition speed on the signal length. The 3960 independent realizations were executed to calculate the average decomposition time with standard deviation for each type of data, as shown in Figure 8. The average decomposition times of sEEMD-CCA for the three types of semi-simulated data were 4.5565 s, 2.9133 s, and 2.0828 s with standard deviations of 0.1349 s, 0.2639 s, and 0.0753 s, respectively, which is shorter than that of EEMD-CCA (9.2715 s, 7.1194 s, and 5.5688 s with standard deviations of 0.2391 s, 0.3030 s, and 0.2280 s, respectively). Whether using the EEMD-CCA or sEEMD-CCA method, as the signal length decreased, the signal decomposition speed increased. For each length of data, the decomposition speed of sEEMD-CCA was faster than that of EEMD-CCA. Compared with the EEMD-CCA method, the running time of the sEEMD-CCA method was reduced by more than 50%. The investigations based on a one-sided *t*-test showed that the difference in signal decomposition speed between sEEMD-CCA and EEMD-CCA was significant (*p* < 0.05). This means that the sEEMD-CCA method significantly improves the running time. Compared with the EEMD-CCA method, the sEEMD-CCA method is well acceptable for separating EMG artifacts from EEG signals in real time.

EMD is an important breakthrough in the field of signal processing, which is widely used in the decomposition of one-dimensional real signals. The algorithm itself has some limitations. For this reason, the derivative algorithms of EMD have been proposed one after another. For example, EEMD is an improvement to the modal aliasing phenomenon of EMD. The complex EMD algorithm realizes the decomposition of complex signal. With the advancement of physics and engineering, the algorithms for synchronous decomposition of multi-dimensional signals are also developed based on EMD.

Multivariate EMD (MEMD) is an extended algorithm of EMD for multi-dimensional data [26]. The algorithm first projects the multi-dimensional signal onto the direction vector of a hypersphere. Then, the envelope in each direction vector is calculated separately. Finally, the mean value of the envelopes is regarded as the local mean value of the multi-dimensional data to successfully realize decomposition of the multi-dimensional data. Compared with EMD and its variants for one-dimensional data, MEMD can more accurately estimate the envelope of the signal by analyzing the inherent modes across multiple channels at the same time instead of channel by channel, so that it can more robustly identify the common activities between multiple channels. Moreover, the IMFs obtained from different EEG channels using EMD and its variants applied to one-dimensional data may differ in order or frequency. MEMD extracts the IMFs with the same order or frequency for the different channels, solving the pattern calibration of multi-channel data. However, it is very difficult to extract the local extrema of multi-dimensional signals to estimate the envelopes in comparison to one-dimensional signals. Therefore, MEMD adopts more complex projection technique and interpolation method to capture the envelopes. The computational cost of these morphological operations is prohibitive. This directly leads to the time-consuming process of envelope identification in each iteration.

In recent years, MEMD has also been introduced to remove artifacts from EEG. Soler et al. [27] used MEMD to separate noise components so as to reconstruct EEG data associated with neural activity. Chen et al. [28] proved that the MEMD-CCA method is a promising tool for removing EMG artifacts from few-channel EEG data. Although the MEMD-CCA method can effectively remove EMG artifacts and preserve EEG information completely, the application of this method is limited by the heavy computational cost of MEMD-CCA (which is much larger than the computational cost of EEMD-CCA). Chen et al. hope that a faster version of MEMD will be released as soon as possible to improve the corresponding situation. Our proposed sEEMD-CCA method not only has a remarkable ability to remove EMG artifacts, but also significantly improved the running speed. Usually, the derivative methods of EMD are improved by optimizing projection techniques and interpolation methods. The sEEMD method provides a new optimization perspective for signal decomposition algorithms, which is based upon changing the structure of the input signal instead of optimizing the projection technique or interpolation method.

This study has some limitations. For example, as there is still a lot of work to be completed to build a real-time BCI system, the effectiveness of the proposed algorithm, the difference in accuracy at the different information transfer rates, and the relationship between the data length, execution time, and accuracy metrics in a real-time BCI system are not discussed in this study. Furthermore, our analyses were only performed on semi-simulated data. The effect of the sEEMD-CCA method on EMG artifact removal from real data was not explored. In future work, we hope that these limitations will be improved.

## 5. Conclusions

EEG is the external manifestation of neural activity inside the brain, which contains rich information reflecting physiology, psychology, and pathology. BCI often uses the information contained in EEG to convert the thoughts of people into real actions. Due to the existence of EMG artifacts, the performance of BCI declines sharply. Therefore, it is necessary to eliminate EMG artifacts in EEG. Existing studies have confirmed that it is effective to remove EMG artifacts employing the EEMD-CCA method. However, the time-consuming iterative process of EEMD may make EEMD-CCA method difficult to match the monitoring requirement of BCI in real time. In order to improve the efficiency of the EEMD-CCA method, a fast method to eliminate EMG artifacts based on sEEMD and CCA was proposed in this study. The results show that the IMFs generated by sEEMD are essentially similar to the IMFs decomposed by EEMD. There was no significant difference in removing EMG artifacts between the sEEMD-CCA method and the EEMD-CCA method (*p* > 0.05). Compared with the EEMD-CCA method, the running speed of sEEMD-CCA to remove EMG artifacts was significantly accelerated (*p* < 0.05). In summary, the sEEMD-CCA method is well-suited to remove EMG artifacts from EEG signals in real time.

## Figures and Tables

**Table 1:** RRMSE between EEG data denoised by each algorithm and clean EEG data under different pollution conditions.

	EEMD-ICA	EEMD-CCA	sEEMD-CCA
SNR = 0.5 dB	0.4945±0.0401	0.4721±0.0387	0.4890±0.0370
SNR = 1 dB	0.4016±0.0491	0.3477±0.0496	0.3568±0.0474
SNR = 1.5 dB	0.3660±0.0587	0.3033±0.0557	0.3020±0.0495
SNR = 2 dB	0.3432±0.0654	0.2782±0.0592	0.2730±0.0514
SNR = 2.5 dB	0.3252±0.0717	0.2615±0.0608	0.2532±0.0515
SNR = 3 dB	0.3088±0.0785	0.2460±0.0658	0.2404±0.0568
SNR = 3.5 dB	0.2952±0.0839	0.2356±0.0709	0.2386±0.0611
SNR = 4 dB	0.2845±0.0864	0.2244±0.0717	0.2325±0.0657
SNR = 4.5 dB	0.2757±0.0875	0.2168±0.0730	0.2300±0.0682
SNR = 5 dB	0.2682±0.0899	0.2122±0.0754	0.2267±0.0689
SNR = 5.5 dB	0.2604±0.0921	0.2071±0.0751	0.2205±0.0700

**Table 2:** Average CC between EEG data denoised by each algorithm and clean EEG data under different pollution conditions.

	EEMD-ICA	EEMD-CCA	sEEMD-CCA
SNR = 0.5 dB	0.8806±0.0241	0.8916±0.0222	0.8902±0.0213
SNR = 1 dB	0.9150±0.0231	0.9359±0.0196	0.9347±0.0197
SNR = 1.5 dB	0.9283±0.0240	0.9502±0.0194	0.9527±0.0175
SNR = 2 dB	0.9362±0.0244	0.9576±0.0184	0.9615±0.0161
SNR = 2.5 dB	0.9421±0.0248	0.9622±0.0178	0.9672±0.0151
SNR = 3 dB	0.9470±0.0253	0.9661±0.0178	0.9700±0.0161
SNR = 3.5 dB	0.9509±0.0257	0.9687±0.0182	0.9702±0.0169
SNR = 4 dB	0.9540±0.0252	0.9716±0.0174	0.9718±0.0174
SNR = 4.5 dB	0.9566±0.0250	0.9733±0.0173	0.9725±0.0174
SNR = 5 dB	0.9587±0.0251	0.9745±0.0172	0.9733±0.0171
SNR = 5.5 dB	0.9608±0.0251	0.9759±0.0166	0.9747±0.0169

## Data Availability

In this study, the EEG dataset employed is the Dataset 2a of BCI Competition IV, which is freely available in [25]. The EMG data are not publicly available but are available from the corresponding author on reasonable request.
